# Symptoms, Sites, and Significance of *Mycoplasma genitalium* in Men Who Have Sex with Men

**DOI:** 10.3201/eid2504.181258

**Published:** 2019-04

**Authors:** Tim R.H. Read, Gerald L. Murray, Jennifer A. Danielewski, Christopher K. Fairley, Michelle Doyle, Karen Worthington, Jenny Su, Elisa Mokany, L.T. Tan, David Lee, Lenka A. Vodstrcil, Eric P.F. Chow, Suzanne M. Garland, Marcus Y. Chen, Catriona S. Bradshaw

**Affiliations:** Melbourne Sexual Health Centre, Carlton, Victoria, Australia (T.R.H. Read, C.K. Fairley, M. Doyle, K. Worthington, D. Lee, L.A. Vodstrcil, E.P.F. Chow, M.Y. Chen, C.S. Bradshaw);; Monash University, Melbourne, Victoria, Australia (T.R.H. Read, C.K. Fairley, L.A. Vodstrcil, E.P.F. Chow, M.Y. Chen, C.S. Bradshaw);; Monash Biomedicine Discovery Institute, Melbourne (G.L. Murray);; Murdoch Children’s Research Institute, Parkville, Victoria, Australia (G.L. Murray, J.A. Danielewski, J. Su, S.M. Garland);; Royal Women’s Hospital, Melbourne (G.L. Murray, J.A. Danielewski, S.M. Garland);; Royal Children’s Hospital, Melbourne (G.L. Murray, S.M. Garland);; SpeeDx Pty Ltd, Eveleigh, Sydney, New South Wales, Australia (E. Mokany, L.T. Tan);; University of Melbourne, Melbourne (S.M. Garland)

**Keywords:** Mycoplasma genitalium, antibiotic resistance, azithromycin, proctitis, men who have sex with men, MSM, Australia, antimicrobial resistance, bacteria, sexually transmitted infections

## Abstract

During 2016–2017, we tested asymptomatic men who have sex with men (MSM) in Melbourne, Australia, for *Mycoplasma genitalium* and macrolide resistance mutations in urine and anorectal swab specimens by using PCR. We compared *M. genitalium* detection rates for those asymptomatic men to those for MSM with proctitis and nongonococcal urethritis (NGU) over the same period. Of 1,001 asymptomatic MSM, 95 had *M. genitalium;* 84.2% were macrolide resistant, and 17% were co-infected with *Neisseria gonorrhoeae* or *Chlamydia trachomatis*. Rectal positivity for *M. genitalium* was 7.0% and urine positivity was 2.7%. *M. genitalium* was not more commonly detected in the rectums of MSM (n = 355, 5.6%) with symptoms of proctitis over the same period but was more commonly detected in MSM (n = 1,019, 8.1%) with NGU. *M. genitalium* is common and predominantly macrolide-resistant in asymptomatic MSM. *M. genitalium* is not associated with proctitis in this population.

*Mycoplasma genitalium* causes nongonococcal urethritis (NGU) in men and is associated with pelvic inflammatory disease (PID), spontaneous abortion, and premature labor in women (*1*,*2*). Most guidelines recommend azithromycin as a first-line treatment; however, macrolide resistance is widespread and increasing in many countries (*3*–*5*). In a recent study of *M. genitalium* urethritis in Melbourne, Victoria, Australia, 39% of cases were in men who have sex with men (MSM); macrolide resistance was detected almost twice as often in MSM as in women or heterosexual men (76% of MSM vs. 39% for women and heterosexual men combined; p = 0.005) (*6*). We hypothesized that this difference may have arisen from frequent treatment of MSM for *Chlamydia trachomatis* and *Neisseria gonorrhoeae* infections, resulting in exposure of asymptomatic *M. genitalium* infections to azithromycin.

*M. genitalium* has been proposed as a cause of proctitis in MSM, but few studies have examined this association. Soni et al. found *M. genitalium* in 4.4% of rectal swabs from 438 MSM in England and found no association with rectal symptoms (*7*). Francis et al. found *M. genitalium* in 5.4% of rectal swabs from 500 MSM in the United States but found only a weak association with rectal symptoms (*8*). Bissessor et al. reported that bacterial load of rectal *M. genitalium* was higher in MSM with proctitis compared with those with asymptomatic infection, and detection was more common in HIV-positive than HIV-negative MSM (21% vs. 8%; p = 0.006) (*9*). A meta-analysis in 2009 of 19 mostly cross-sectional or case–control studies found an association between *M. genitalium* and HIV infection, particularly in studies from sub-Saharan Africa (*10*). Subsequently, *M. genitalium* was detected twice as commonly in women who seroconverted to HIV in a prospective study in Africa (*11*), but no equivalent studies in MSM are available.

We aimed to determine the proportion of asymptomatic MSM who had *M. genitalium* in the urethra or rectum and the prevalence of macrolide resistance and risk factors for infection. We compared these data with the proportion of tests positive for *M. genitalium* in MSM with symptoms of proctitis and nongonococcal urethritis to further examine the contribution of *M. genitalium* to these syndromes in MSM.

## Methods

This cross-sectional study was undertaken during August 23, 2016–September 27, 2017, at Melbourne Sexual Health Centre (MSHC), the only public sexual health clinic in Melbourne, a city of 4.5 million. MSM >18 years of age who were asymptomatic at both triage and clinician consultations and reported receptive anal sex within the preceding year were eligible to participate. To minimize the impact of this study on clinical and laboratory services, recruitment was restricted to 8 of 49 clinical staff members, who offered the study to consecutive eligible clients. To determine how representative participants were of all asymptomatic MSM attending MSHC, we compared positivity for rectal *C. trachomatis* and *N. gonorrhoeae* in recruited and nonrecruited MSM. We asked participants to complete a questionnaire about recent sexual risk practices and to record any anogenital symptoms experienced in the preceding week. Participants provided urine and a rectal swab specimen (self- or clinician-collected) for *M. genitalium* screening. 

We agitated the rectal swabs in 0.6 mL of phosphate-buffered saline to release cellular material, vortexed them briefly, and centrifuged them at low speed (8,000 rcf, 10 min) to remove PCR inhibitors. This step was required to reduce inhibition that differentially affected rectal samples; in early evaluations, the internal control failed in 9 (20.5%) of 44 uncentrifuged rectal samples but in none of 106 samples subjected to centrifugation. We transferred 0.2 mL of supernatant for nucleic acid isolation using the MagNA Pure 96 DNA and viral small volume kit on the automated MagNA Pure 96 system (Roche Diagnostics, https://www.roche.com). We prepared urine samples as described previously (*12*). We detected *M. genitalium* and macrolide resistance mutations in the 23S rRNA gene using the ResistancePlus MG test (SpeeDx Pty Ltd, Australia, https://plexpcr.com). Published evaluations of this assay report specificity for the detection of *M. genitalium* of 100% and sensitivities of 94.9%, 98.5%, and 98.9% (*13*–*15*).

Participants provided additional samples for *C. trachomatis* and *N. gonorrhoeae* screening of the throat, urethra, and rectum; we performed serologic testing for syphilis and HIV as indicated. We tested samples for *N. gonorrhoeae* and *C. trachomatis* by transcription-mediated amplification (Aptima Combo 2, Hologic, https://www.hologic.com).

MSM who were recalled for treatment of *M.genitalium* completed another questionnaire about antimicrobial drug use. We also collected throat swab specimens from men with rectal *M. genitalium* infection so we could perform pharyngeal *M. genitalium* testing. Resources were not available for testing all participants, particularly since published studies have rarely detected *M. genitalium* at this site. However, we hypothesized that *M. genitalium* may be more common in the pharynx in men with rectal *M. genitalium*. We agitated the throat swabs in 0.6 mL of phosphate-buffered saline to release cellular material and performed nucleic acid isolation as described for the other samples.

### Statistical Methods

With a sample size of 1,000, a prevalence of *M. genitalium* of 10% would provide 80% power (α = 0.05) to detect an odds ratio of >1.9 for a characteristic present in 30% of those who did not have *M. genitalium*. We assessed associations between *M. genitalium*, *C. trachomatis*, and *N. gonorrhoeae* and risk factors, as well as mild urethral and anorectal symptoms reported in the questionnaire, using logistic regression.

All patients attending MSHC who have symptoms of nongonococcal urethritis or proctitis are tested for *M. genitalium.* During the 13-month study period, we also extracted test results from the clinic database for *M. genitalium*, *C. trachomatis*, and *N. gonorrhoeae* from MSM who received diagnoses of proctitis or urethritis (based on symptoms and signs, not microscopic criteria). Using univariate logistic regression, we then used corresponding test results from the asymptomatic study population as controls to assess any association between detection of each organism in the rectum and urine and diagnoses of proctitis and urethritis. For men with *M. genitalium* detected, we compared risk factors for macrolide resistance mutations using χ^2^ or Fisher exact tests, where appropriate. We also recorded the proportions of *M. genitalium* patients co-infected with *C. trachomatis* or *N. gonorrhoeae* in the urethra and rectum. We compared associations between the detection of *M. genitalium* and that of *C. trachomatis* or *N. gonorrhoeae* in the rectum or urine in the asymptomatic study population using logistic regression, as we did with associations between *M. genitalium* and *C. trachomatis* in cases of nongonococcal urethritis diagnosed during the same period.

This project was approved by the ethics committee of the Alfred Hospital in Melbourne (project no. 278/16). All participants gave written informed consent.

## Results

During August 23, 2016–September 27, 2017, a total of 1,028 MSM were triaged as asymptomatic and invited to participate in the study. Of these, 17 declined: 3 declined the additional rectal swab specimen collection, and 14 declined for reasons unrelated to the study (distress or being unable to return to the clinic). Of the remaining 1,011, a total of 6 rectal swabs were unassessable (internal control failed), and 4 did not provide a urine sample. These 10 patients were excluded from the analysis, leaving 1,001 men with assessable samples from both collection sites.

Participants had a median age of 28.8 (interquartile range 24.3–34.1). A total of 107 (10.7%) were HIV positive, and 142 (15.9%) of the HIV-negative men were taking or commencing HIV preexposure prophylaxis medication (PrEP) (Table 1).

**Table 1:** Characteristics associated with urethral or rectal *Mycoplasma genitalium* in asymptomatic men who have sex with men, Australia*

Characteristic	All patients	*M. genitalium* not detected	*M. genitalium* detected†	Crude OR (95% CI)	p value
Detected in urine, rectum, or both					
Prevalence	1,001	906 (90.5)	95 (9.5, 7.7–11.5)		
Median age, y (IQR)	28.8 (24.3–34.1)	28.9 (24.5–34.3)	27.4 (23.3–32.3)	0.96 (0.93–0.99)	0.006
HIV status‡					
Negative	894	804 (88.7)	90 (94.7)		
Positive	107	102 (11.3)	5 (5.3, 1.7–11.9)	0.44 (0.17–1.10)	0.08
On/commencing PrEP§					
No	752	678 (84.3)	74 (82.2)		
Yes	142	126 (15.7)	16 (17.8, 10.5–27.3)	1.16 (0.66–2.06)	0.60
Detected in urine only					
Urine prevalence		974 (97.3)	27 (2.7, 1.8–3.9)		
Insertive oral sex partners in previous 3 mo, n = 984¶					
<4	431	421 (44.0)	10 (37.0)		
>4	553	536 (56.0)	17 (63.0)	1.34 (0.61–2.95)	0.47
Insertive anal sex partners in previous 3 months, n = 941#					
<2	428	418 (45.7)	10 (38.5)		
>2	513	497 (54.3)	16 (61.5)	1.34 (0.60–3.0)	0.47
Condom use insertive anal sex in previous 3 mo					
Always	287	280 (38.7)	7 (29.2)		
Not always	460	443 (61.3)	17 (70.8)	1.53 (0.63–3.75)	0.35
Detected in rectum only					
Rectal prevalence		931 (93.0)	70 (7.0, 5.5–8.8)		
Receptive anal sex partners in previous 3 mo, n = 945#					
<2	367	349 (39.8)	18 (26.1)		
>2	578	527 (60.2)	51 (73.9)	1.88 (1.08–3.3)	0.026
Condom use receptive anal sex in previous 3 mo					
Always	301	288 (37.1)	13 (20.0)		
Not always	540	488 (62.9)	52 (80.0)	2.36 (1.24–4.81)	0.006

*Values are no. (%, 95% CI) except as indicated. This table should be viewed in conjunction with Table 2. IQR, interquartile range; OR, odds ratio; PrEP, preexposure prophylaxis. †In 2 of 97 infected men, *M. genitalium* was detected in both the urine and the rectum. ‡Includes 5 men with unknown HIV infection status. *M. genitalium* was detected in 4.7% of HIV-positive men vs. 10.1% of HIV-negative men (p = 0.08). §HIV-negative men only. ¶Median 4. #Median 2.

Of the 1,001 men, 95 (9.5% [95% CI 7.7%–11.5%]) had *M. genitalium* detected at any site. Twenty-seven (2.7% [95% CI: 1.8%–3.9%]) had *M. genitalium* detected in the urine and 70 (7.0% [95% CI 5.5%–8.8%]) in the rectum; 2 men were infected at both sites. *C. trachomatis* was detected in 91 (9.6% [95% CI 7.8%–11.7%]) of 948 men tested at both sites, and *N. gonorrhoeae* was detected in 64 (6.7% [95% CI 5.2%–8.5%]) of 952 men tested at both sites (Table 2). For urine samples, *M. genitalium* was detected in 2.7%, *C. trachomatis* in 1.7%, and *N. gonorrhoeae* in 0.7%: For rectal samples, *M. genitalium* was detected in 7.0%, *C. trachomatis* in 8.5%, and and *N. gonorrhoeae* in 6.2%.

**Table 2:** Detection of urethral or rectal *Chlamydia trachomatis* or *Neisseria gonorrhoeae* in asymptomatic men who have sex with men, Australia*

Characteristic	All patients	*C. trachomatis* not detected	*C. trachomatis* detected†	*N. gonorrhoeae* not detected	*N. gonorrhoeae* detected†
Detected in urine, rectum, or both					
STI prevalence	1,001	857 (90.4)	91 (9.6, 7.8–11.7)	888 (93.3)	64 (6.7, 5.2–8.5)
Median age, y (IQR)	28.8 (24.3–34.1)	28.8 (24.3–34.0)	27.6 (23.8–35.2)	28.8 (24.3–34.1)	27.2 (24.1–33.1)
HIV status					
Negative‡	894	782 (91.3)	72 (79.1)	801 (90.2)	55 (85.9)
Positive	107	75 (8.8)	19 (20.9)	87 (9.8)	9 (14.1)
On/commencing PrEP§					
No	752	666 (85.2)	57 (79.2)	683 (85.3)	39 (70.9)
Yes	142	116 (14.8)	15 (20.8)	118 (14.7)	16 (29.1)
Detected in urine only					
Urine prevalence	958	942 (98.3)	16 (1.7, 1.0–2.7)	951 (99.3)	7 (0.7, 0.3–1.4)
Insertive oral sex partners in previous 3 mo, n = 984¶					
<4	431	407 (44.0)	4 (25.0)	408 (43.6)	2 (28.6)
>4	553	519 (56.0)	12 (75.0)	527 (56.4)	5 (71.4)
Insertive anal sex partners in previous 3 mo, n = 941#					
<2	428	406 (45.9)	4 (25.0)	409 (45.8)	0
>2	513	479 (54.1)	12 (75.0)	485 (54.2)	7 (100)
Condom use insertive anal sex in previous 3 mo					
Always	287	273 (39.1)	5 (31.3)	273 (38.6)	4 (57.1)
Not always	460	425 (60.9)	11 (68.7)	434 (61.4)	3 (42.9)
Detected in rectum only					
Rectal prevalence	958–963	877 (91.5)	81 (8.5, 6.8–10.4)	903 (93.8)	60 (6.2, 4.8–7.9)
Receptive anal sex partners in previous 3 mo, n = 945#					
<2	367	336 (40.8)	20 (24.7)	342 (40.1)	15 (26.3)
>2	578	488 (59.2)	61 (75.3)	511 (59.9)	42 (73.7)
Condom use receptive anal sex in previous 3 mo					
Always	301	270 (37.1)	18 (23.4)	277 (36.7)	11 (20.0)
Not always	540	458 (62.9)	59 (76.6)	477 (63.3)	44 (80.0)

*Values are no. (%) or no. (%, 95% CI) except as indicated. This table should be viewed in conjunction with Table 1. All 1,001 men had urine and rectal swabs tested for *Mycoplasma genitalium*, but only 948 were tested at both sites for *C. trachomatis* and 952 for *N. gonorrhoeae.* IQR, interquartile range; PrEP, preexposure prophylaxis. †Denominators varied based on numbers tested; 958 men had urine tests for both infections; 948 men were screened at both sites and 958 men had rectal tests for *C. trachomatis*; and 952 men were screened at both sites and 963 men had rectal tests for *N. gonorrhoeae*. ‡Includes 5 men of unknown HIV infection status. *M. genitalium* was detected in 4.7% of HIV-positive men vs. 10.1% of HIV-negative men (p = 0.08). §HIV-negative men only. ¶Median 4. #Median 2.

During the study period, 4,228 MSM were triaged as asymptomatic at MSHC and not offered the study but were tested for rectal *C. trachomatis* and *N. gonorrhoeae* at least once. After excluding repeat tests, positivity for *C. trachomatis* did not differ between nonrecruited (7.4%) and recruited (8.5%) MSM (p = 0.25), but *N. gonorrhoeae* was lower in nonrecruited (4.2%) than in recruited (6.2%) MSM (p = 0.006).

Detection of *M. genitalium* was significantly associated with younger age (odds ratio [OR] 0.96 [95% CI 0.93–0.99]) per year of increasing age. Detection of *M. genitalium* in the rectum was significantly associated with receptive anal sex with >2 partners within the past 3 months (OR 1.88 [95% CI 1.08–3.3]) and inconsistent condom use for receptive anal sex (OR 2.36 [95%CI 1.24–4.81]). *M. genitalium* was less common in HIV-infected men than in uninfected men (4.7% vs 10.1%, p = 0.08) but was not associated with taking or commencing PrEP.

The study population of 1,001 asymptomatic MSM completed a questionnaire about the presence of any anogenital or urethral symptoms in the week before presentation (all participants were asymptomatic at recruitment). Of these, 8.7% reported any recent symptoms in the urethra (itch, discomfort, discharge, or dysuria) and 25.5% in the anorectum (itch, discomfort, pain, or bleeding). Recent symptoms was not associated with detection of *M. genitalium*, *C. trachomatis*, or *N. gonorrhoeae* at either site (p>0.5 for all symptoms, individually or combined; Table 3).

**Table 3:** Detection of *Mycoplasma genitalium*, *Chlamydia trachomatis*, and *Neisseria gonorrhoeae* in asymptomatic men who have sex with men according to reports of symptoms during the preceding week, Australia*

Characteristic	Urethral symptoms†					Anorectal symptoms‡			
	None, no. (%)	Mild, no. (%)	Odds ratio (95% CI)	p value		None, no. (%)	Mild, no. (%)	Odds ratio (95% CI)	p value
*M. genitalium*, n = 1,001									
Not detected	889 (97.3)	85 (97.7)				692 (92.7)	239 (93.7)		
Detected	25 (2.7)	2 (2.3)	0.84 (0.19–3.59)	0.81		54 (7.2)	16 (6.3)	0.86 (0.48–1.53)	0.60
*C. trachomatis*, n = 958									
Not detected	861 (98.4)	81 (97.6)				657 (91.4)	220 (92.1)		
Detected	14 (1.6)	2 (2.4)	1.52 (0.34–6.80)	0.59		62 (8.6)	19 (7.9)	0.92 (0.54–1.56)	0.75
*Neisseria gonorrhoeae*, n = 958									
Not detected	868 (99.3)	83 (98.8)				675 (93.8)	228 (93.8)		
Detected	6 (0.7)	1 (1.2)	1.74 (0.21– 14.65)	0.61		45 (6.3)	15 (6.2)	0.99 (0.54–1.80)	0.97

*All participants were triaged as asymptomatic. This table reports answers to a questionnaire about “any symptoms (even if mild) in the past week.” †Urethral symptoms were any of the following: dysuria, discharge, urethral itch, or discomfort. No individual symptom was significantly associated with any organism. ‡Anorectal symptoms were any of the following: anal pain, bleeding, itch, or discomfort. No individual symptom was significantly associated with any organism.

We compared rectal test positivity for *M. genitalium*, *C. trachomatis*, and *N. gonorrhoeae* in the asymptomatic study population (n = 1,001) with rectal positivity in MSM who had symptoms of proctitis (n = 355) during the study period (Table 4). *M. genitalium* detection was similar in MSM with proctitis and asymptomatic MSM (5.6% for proctitis vs. 7.0% for asymptomatic; OR 0.79 [95% CI 0.45–1.35]; p = 0.38). However, rectal detection of both *C. trachomatis* (21.3% vs. 8.5%, OR 2.93 [95% CI 2.05–4.18]) and *N. gonorrhoeae* (28.4% vs. 6.2%, OR 5.97 [95% CI 4.15–8.61]) were significantly more common in MSM with symptoms of proctitis than in asymptomatic MSM.

**Table 4:** Detection of *Mycoplasma genitalium*, *Chlamydia trachomatis*, and *Neisseria gonorrhoeae* in asymptomatic men who have sex with men compared with clinic attendees diagnosed with proctitis or NGU, Australia*

Characteristic	Asymptomatic men tested at the rectum for STIs	Clinic attendees with symptoms of proctitis	Odds ratio (95% CI)	p value	Asymptomatic men tested at the urethra for STIs	Clinic attendees with symptoms of NGU	Odds ratio (95% CI)	p value
*M. genitalium*								
Not detected	931 (93.0)	335 (94.4)			974 (97.3)	936 (91.9)		
Detected	70 (7.0)	20 (5.6)	0.79 (0.45–1.35)	0.38	27 (2.7)	83 (8.1)	3.20 (2.03–5.18)	<0.0001
*C. trachomatis*								
Not detected	877 (91.5)	277 (78.7)			942 (98.3)	878 (85.5)		
Detected	81 (8.5)	75 (21.3)	2.93 (2.05–4.18)	<0.0001	16 (1.7)	149 (14.5)	9.99 (5.89– 18.07)	<0.0001
*N. gonorrhoeae*								
Not detected	903 (93.8)	252 (71.6)						
Detected	60 (6.2)	100 (28.4)	5.97 (4.15–8.61)	<0.0001				

*Treated as NGU but not confirmed by urethral Gram stain. NGU, nongonococcal urethritis; STI, sexually transmitted infection.

We compared the urine test positivity for *M. genitalium* and *C. trachomatis* in the asymptomatic study population (n = 1,001) with the positivity in 1,019 MSM presenting with symptoms of NGU during the study period. Both *M. genitalium* (8.1% vs. 2.7%; OR 3.20 [95% CI 2.03–5.18]) and *C. trachomatis* (14.5% vs. 1.7%, OR 9.99 [95% CI 5.89–18.07]) were more commonly detected in MSM with symptoms of NGU than in asymptomatic MSM (Table 4).

We detected macrolide resistance mutations in 80 (84.2% [95% CI 75.3%–90.9%]) of 95 men who had positive *M. genitalium* tests (Table 5). We found no significant association between resistance and site of infection, and although these mutations were more common in MSM reporting recent use of antimicrobial drugs, particularly azithromycin, this difference was not significant. Macrolide resistance mutations were found in all HIV-negative men taking or commencing PrEP (p = 0.06).

**Table 5:** Risk factors for detection of macrolide resistance mutations in anogenital *Mycoplasma genitalium* infections detected in men who have sex with men, Australia

Category	Mutations not detected, no. (%)	Mutations detected, no. (%)	p value
Overall	15 (15.8)	80 (84.2)	
Antibiotic in the preceding 3 months			
None	13 (86.7)	54 (67.5)	
Yes, not azithromycin	2 (13.3)	17 (21.3)	
Yes, azithromycin	0	9 ( 11.2)	0.39
HIV status			
Negative	15 (100.0)	75 (93.8)	
Positive	0	5 ( 6.2)	1.0
Taking or starting PrEP*			
No	15 (100.0)	59 (78.7)	
Yes	0	16 (21.3)	0.06
Site of infection			
Urine	4 (26.7)	23 (28.0)	
Rectum	11 (73.3)	59 (72.2)	0.91

*PrEP, preexposure prophylaxis. HIV-positive men excluded.

Table 6 shows the proportion of asymptomatic MSM with *M. genitalium* who were co-infected with *C. trachomatis* and *N. gonorrhoeae*, by anatomic site. Rectal *C. trachomatis* and rectal *N. gonorrhoeae* were detected with similar frequency in MSM with rectal *M. genitalium* compared with men without rectal *M. genitalium* (*C. trachomatis*, 9.2% vs. 8.4%, p = 0.82; *N. gonorrhoeae*, 6.1% vs. 6.2%; p = 0.98). However, *C. trachomatis* and *N. gonorrhoeae* were detected significantly more often in the urine of asymptomatic men with *M. genitalium* compared with men without urethral *M. genitalium* (*C. trachomatis*, 7.4% vs. 1.5%, p = 0.03; *N. gonorrhoeae*, 7.4% vs. 0.5%, p = 0.002).

**Table 6:** *Mycoplasma genitalium* detection in men who have sex with men co-infected with *Chlamydia trachomatis* or *Neisseria gonorrhoeae* and are asymptomatic or have symptoms of NGU, Australia*

Category	Rectal *M. genitalium*		Odds ratio (95% CI)	p value
	Not detected, no. (%)	Detected, no. (%)		
Asymptomatic, n = 1,001				
Rectal *C. trachomatis*				
Not detected	818 (91.6)	59 (90.8)		
Detected	75 (8.4)	6 (9.2)	1.10 (0.46–2.65)	0.82
Rectal *N. gonorrhoeae*				
Not detected	842 (93.8)	61 (93.9)		
Detected	56 (6.2)	4 (6.1)	0.96 (0.35–2.81)	0.98
	Urine *M. genitalium*			
Urine *C. trachomatis*				
Not detected	917 (98.5)	25 (92.6)		
Detected	14 (1.5)	2 (7.4)	5.24 (1.13–24.29)	0.03
Urine *N. gonorrhoeae*				
Not detected	926 (99.5)	25 (92.6)		
Detected	5 (0.5)	2 (7.4)	14.82 (2.74–80.07)	0.002
Men with NGU symptoms,† n = 1,001*	Urine *M. genitalium*			
Urine *C. trachomatis*				
Not detected	777 (84.5)	79 (97.5)		
Detected	143 (15.5)	2 (2.5)	0.14 (0.03–0.57)	0.001

*Although there are 1,001 men in each dataset, these 2 groups are the same size only by coincidence. NGU, nongonococcal urethritis. †All men in this group received a clinical diagnosis of urethritis.

In contrast, in MSM with NGU, detection of *C. trachomatis* was uncommon in men with urethral *M. genitalium* (2.5%) compared with men without urethral *M. genitalium* (15.5%; p = 0.001). Overall, of 89 MSM with *M. genitalium* infection detected at any site and tested for all 3 infections, 15 (16.9% [95% CI 9.7–26.3]) were co-infected with either *C. trachomatis* or *N. gonorrhoeae.* Of 143 MSM with either *C. trachomatis* or *N. gonorrhoeae*, 15 (10.5% [95% CI 5.9%–16.7%]) were co-infected with *M. genitalium.*

Throat swabs were collected from 54 (77.1%) of 70 MSM with rectal *M. genitalium*, all 60 MSM with rectal *N. gonorrhoeae*, and 37 (45.7%) of 81 MSM with rectal *C. trachomatis* (routine clinic testing for pharyngeal *C. trachomatis* commenced halfway through the study). Only 1 (1.9% [95% CI 0.05–9.9]) of 54 MSM with rectal *M. genitalium* had pharyngeal *M. genitalium*. In contrast, 8 (21.6% [95% CI 9.8–38.2]) of 37 MSM with rectal chlamydia had pharyngeal chlamydia, and 21 (35% [95% CI 23.1–48.4]) of 60 MSM with rectal gonorrhea had pharyngeal gonorrhea. Thus, dual pharyngeal and rectal infection with *M. genitalium* was significantly less common than was observed for *C. trachomatis* (p = 0.002) and *N. gonorrhoeae* (p<0.001). Of all men tested, 12 (3.0%) of 407 had pharyngeal chlamydia and 62 (6.4%) of 963 had pharyngeal gonorrhea.

## Discussion

Almost 1 in 10 asymptomatic MSM attending a sexual health center in Melbourne, Victoria, Australia, during a 13-month period were infected with *M. genitalium*, and 84% of these infections were macrolide resistant. *M. genitalium* was detected in 7% of asymptomatic MSM at the rectum, 2.7% at the urethra, and only 0.2% at both sites. Overall, *M. genitalium* was as common as chlamydia and more common than gonorrhea in asymptomatic MSM. The proportion of asymptomatic MSM with *M. genitalium* in the rectum was no different from that in MSM with symptoms of proctitis during the same period. Co-infection with *C. trachomatis* or *N. gonorrhoeae* was common and present in 17% of *M. genitalium* infections. Screening MSM for *C. trachomatis* and *N. gonorrhoeae* will identify these infections, but if they are treated, asymptomatic *M. genitalium* infections, present in 10% of these cases, may be inadvertently exposed to azithromycin, exerting selection pressure for macrolide resistance, which may explain the rapid escalation of resistance in *M. genitalium* in MSM. The situation facing clinicians is challenging, because the recommended treatment for macrolide-resistant *M. genitalium*, moxifloxacin, is expensive, potentially toxic, and difficult to obtain and may generate further antimicrobial resistance, all of which should be considered before screening asymptomatic MSM for *M. genitalium.*

The detection of *M. genitalium* in 9.5% of asymptomatic MSM contrasts with a recent meta-analysis finding lower average prevalence estimates among MSM of 3.2% (95% CI 2.1%–5.1%) in 5 community-based studies and 3.7% (95% CI 2.4%–5.6%) in 4 clinic-based studies (*16*). This discrepancy may be because the meta-analysis included several studies that tested only urine, where *M. genitalium* is less common, or because of geographic or temporal differences. A recent Sydney study reported *M. genitalium* in 13.4% of MSM (rectum 8.9%, urine 4.7%) (*17*).

Rectal positivity for *M. genitalium* in men with symptoms of proctitis was no higher than in asymptomatic MSM. Furthermore, a report of mild anorectal symptoms over the preceding week was not associated with rectal *M. genitalium* and presumably reflected nonspecific self-limiting symptoms. In contrast, rectal *C. trachomatis* and *N. gonorrhoeae* were significantly associated with current symptoms of proctitis (OR 3 and 6, respectively). Two previous studies found no association between rectal *M. genitalium* and symptoms, whereas 1 reported a weak association of borderline significance (*7*,*8*,*17*). Other studies suggesting that *M. genitalium* may cause proctitis have not compared frequency of detection in symptomatic and asymptomatic patients (*18*,*19*).

The high proportion of cases with macrolide resistance in this study (84%) is consistent with recent MSHC data. MSHC has been using the same resistance assay for *M. genitalium* since June 20, 2016; by March 27, 2018, a total of 943 patients with NGU, cervicitis, PID, proctitis, or contacts of infection had *M. genitalium* detected. Macrolide resistance mutations were routinely detected in 265 (51.5% [95% CI 47.0–55.9]) of 515 heterosexual men and women compared with 349 (81.5% [95% CI 77.5–85.1]) of 428 MSM (p<0.0001). This difference between MSM and heterosexuals was also seen in a recent study in Spain, which reported macrolide resistance in 71% of MSM compared with 13% of heterosexuals (p<0.001); prior azithromycin exposure was a significant risk factor for resistance (*20*). Other recent studies in MSM report macrolide resistance in 74%–80% of *M. genitalium* infections (*17*,*21*). The high proportion of cases with resistance reduced our ability to identify risk factors; we detected resistance in 90% of infected men who recalled taking any antimicrobial drug within the previous 3 months and 100% of those who recalled taking azithromycin, but this difference was not significant.

Asymptomatic urethral co-infections with *C. trachomatis* and *N. gonorrhoeae* were significantly associated with detection of *M. genitalium*, but this association was not seen with rectal co-infections. Although the association between *M. genitalium* and urethral co-infections was significant, we found only 4 cases of co-infection. Specific host factors might possibly lead some men to tolerate, and therefore accumulate, urethral infections. The proportion of asymptomatic men with *M. genitalium* detected in their urine was higher than for *C. trachomatis* and for *N. gonorrhoeae*, again consistent with the hypothesis that *M. genitalium* might be less pathogenic than *C. trachomatis* or *N. gonorrhoeae*.

Pharyngeal *M. genitalium* is reported as rare (*22*–*26*), so to optimize detection, we limited pharyngeal testing to MSM with rectal infection because other pharyngeal sexually transmitted infections (STIs) are commonly concurrent with rectal infections. Of patients with rectal *M. genitalium*, only 1.9% had pharyngeal *M. genitalium*, which was much lower than for pharyngeal *C. trachomatis* (22%) and *N. gonorrhoeae* (35%) in MSM with these rectal infections. However, *C. trachomatis* and *N. gonorrhoeae* were detected by transcription mediated amplification. A recent Sydney study using the ResistancePlus PCR assay also found no pharyngeal *M. genitalium* infections in 508 MSM (rectal prevalence 8.9%), providing further evidence that *M. genitalium* is rarely detected in pharyngeal specimens (*17*).

Of concern, 17% of MSM with *M. genitalium* were co-infected with *C. trachomatis* or *N. gonorrhoeae*, predominantly reflecting rectal infections. The rectum appears likely to be a reservoir for asymptomatic *M. genitalium*, and treatment of concurrent STIs promotes macrolide resistance, which is estimated to develop de novo in 12% of wild-type cases exposed to single-dose azithromycin (*6*). The high proportion of macrolide-resistant *M. genitalium* in MSM may be caused by the combination of a high background prevalence of asymptomatic rectal *M. genitalium*, a high frequency of concurrent chlamydia or gonorrhea, and the resulting use of azithromycin in this population.

This study has limitations, including reliance on recall of antimicrobial drug exposure, recruitment from a sexual health center where findings may not reflect MSM elsewhere, and restricted testing for pharyngeal *M. genitalium*. Centrifugation to remove PCR inhibitors was undertaken on rectal samples because of higher levels of inhibition, which could have reduced the sensitivity of rectal *M. genitalium* detection. Furthermore, we were unable to approach all MSM attending the clinic. The study population had a higher proportion with rectal gonorrhea, but not chlamydia, compared with those who were not recruited, possibly because our inclusion criteria required receptive anal sex in the previous year and the nonrecruited group included MSM attending an express service for lower-risk men. This difference suggests that the study population may have had a slightly elevated risk of infection, which should be considered alongside our findings. Diagnoses of nongonococcal urethritis and proctitis were predominantly clinical, based on symptoms and sexual risk, which is likely to lead to a lower prevalence of STIs in these groups compared with studies that required microscopic criteria for case definitions. However, clinical diagnoses are commonly used in primary care and are supported by the strong associations we observed between detection of *C. trachomatis* and *N. gonorrhoeae* and the symptom-based definitions of proctitis and urethritis.

We detected *M. genitalium* in 9.5% of asymptomatic MSM; although it was as common as chlamydia or gonorrhoea, 84% of these infections were macrolide resistant. The high proportion of MSM with macrolide-resistant *M. genitalium* might be considered a reason to screen for this infection but would not meet the criteria for screening established by Wilson and Jungner (*27*). For example, the natural history of *M. genitalium* infection, particularly in the rectum, is poorly understood. Testing is not widely available and the high prevalence of antimicrobial drug resistance also limits the availability of treatment. If we screened this population, 8% of MSM (84% of 9.5%) would require moxifloxacin or a similar agent. Moxifloxacin is expensive, difficult to obtain in many parts of the world, and associated with uncommon but concerning toxicities. Resistance to quinolone antimicrobial drugs is now detected in 16% of patients coming to MSHC (mixed heterosexual and MSM population) in ongoing unpublished work (G.L. Murray, unpub. data). Increasing the use of moxifloxacin as a result of screening would be expected to generate more resistance.

Rectal *M. genitalium* infection may not warrant treatment. It was not associated with current anorectal symptoms in this study; most published literature suggests no association or only a weak association. No prospective studies have associated *M. genitalium* with increased risk for HIV infection in MSM, in contrast to women; such an association may become less critical when HIV PrEP and treatment are widely used. Therefore, screening asymptomatic MSM for *M. genitalium* would result in considerable expense and adverse events for uncertain benefit. Although *M. genitalium* has been identified in cases of proctitis, it is predominantly asymptomatic in the rectum, and there appears to be insufficient evidence to suggest that *M. genitalium* is a cause of proctitis.
